# Determination of the Elasticity Modulus of Additively Manufactured Wrist Hand Orthoses

**DOI:** 10.3390/ma13194379

**Published:** 2020-10-01

**Authors:** Krzysztof Łukaszewski, Radosław Wichniarek, Filip Górski

**Affiliations:** Faculty of Mechanical Engineering, Poznan University of Technology, Piotrowo 3 STR, 61-138 Poznan, Poland; radoslaw.wichniarek@put.poznan.pl

**Keywords:** wrist-hand orthosis, fused filament fabrication, finite element analysis, experimental testing, material properties

## Abstract

The article describes the experimental and simulation research on the material properties of the individualized wrist orthoses produced in the additive manufacturing (AM) process by the fused filament fabrication (FFF) method. The authors produced a series of standard (normalized) samples for three-point bending from acrylonitrile butadiene styrene (ABS) filament on a low-budget 3D printer and a series of samples in the shape of a fragment of the orthosis and the entire orthosis. All types of samples were subjected to experimental tests on a universal testing machine, which allowed us to determine the modulus of elasticity of the produced materials by comparing it with finite element method (FEM) simulation models in the ABAQUS environment. The adopted research methodology allowed us to compare the material properties of the material of the entire product—wrist hand orthosis (WHO)—with the material properties of standard bending samples. The obtained values of Young’s modulus are characterized by a large discrepancy between the standard samples and the entire orthosis. On the other hand, the samples with the shape of the middle part of the orthosis were similar in the value of Young’s modulus to the results obtained during the examination of the complete orthosis.

## 1. Introduction

Nowadays, it is more and more common to use products created in the process of additive manufacturing in layers (additive manufacturing, AM). The fused deposition modeling (FDM)/fused filament fabrication (FFF) method, which is popularly referred to as 3D printing using thermoplastics, is one of the most popular AM methods. Additively manufactured products have various applications including medical ones, for example, mid-surgery supplies [1], implants and endoprostheses [2] or individualized limb prostheses and orthoses (e.g., WHO, wrist-hand orthosis) with an openwork structure [3].

Products manufactured using the FDM/FFF method are made by deposing layers of a plasticized thermoplastic material [4], in the form of a wire, known popularly as a filament. The most commonly used materials are acrylonitrile butadiene styrene (ABS) and polylactic acid (PLA) thermoplastics, but the range of materials and their applications is constantly growing [5].

Products manufactured by this technique are characterized by high anisotropy of strength properties, and the processing conditions significantly affect the values of the technical coefficients of used materials. Altering merely a single process parameter—product orientation in the working space—associated with the application of material layers by FDM/FFF devices, can make the material properties between individual products differ significantly [6]. In [7], the authors indicated that for parts produced additively, although they are formed layer by layer, a different approach to analytical determination of product properties is necessary, rather than the assumed models for laminated composites developed so far. The authors in [8] proposed that a product manufactured using the FDM/FFF method should be analyzed as an orthotropic composite, in which the extruded material fibers constitute the matrix, and the filling are voids, which were not filled with the material during the manufacturing process. Comparison of two analytical models designed using ABAQUS software confirmed that FDM/FFF products should be treated as orthotropic materials. The solid and layered models allowed results similar to tests on real samples to be obtained [9]. An analytical model for multi-material WHO manufactured in a single FDM/FFF process with a dual extruder machine will be an even bigger challenge [10].

An equally important issue when considering the properties of products manufactured by the FDM/FFF method, indirectly related to many process parameters and product geometry, is the temperature aspect of the preceding layer at the time of applying the next layer. The authors of the work [11] showed that the sublayer temperature is important for the UTS (ultimate tensile strength) value. The temperature value depends on many parameters including the temperature of the bed, head, or printing speed. For small-sized samples, the extruded material from the preceding layer will have less time to cool down than for a product with large dimensions. Therefore, it is reasonable to suppose that a similar relationship may appear in the case of other indicators of the strength properties assessment of products manufactured with the FDM/FFF method. The work in [11] also showed that the extrusion efficiency had a significant influence on the strength parameters, and more precisely, the presence of the so-called volume errors related to the nature of the FDM/FFF process. These are the locations where the material has not been deposed. Each such space changes the final density of the entire product. In a very simplified way, it can be assumed that products of similar density should have similar properties, but it is also important in what locations and in what form these volume errors occur in a given product. Nevertheless, by measuring the mass of geometrically identical samples from a particular series, it can be used to infer the repeatability of the manufacturing process.

The FDM/FFF technique is characterized by the fact that in the case of complex geometry (e.g., shape of openwork holes), it may be necessary to use support materials. Supports are auxiliary material supporting the product material above it [12]. They not only extend the production time, because they must be manufactured using the same FDM/FFF device, but also after the end of the process they must be removed, usually mechanically, using simple workshop tools. The use of supports is also associated with a greater consumption of material, and therefore the cost of making the product, which may be important in medical applications where the cost and time of waiting for the patient or doctor to the product is sought. Another disadvantage is the possibility of damage to the product during removal of the support, if there is no possibility of using a dissolvable support, which can be the case when using a single-nozzle machine, or when the processing parameters of used build material (e.g., PA12, PEEK) does not match any of the available dissolvable support materials.

Additive manufacturing FDM/FFF allows for a fully individualized, single piece of product in a relatively short time to be obtained, compared to traditional production techniques (e.g., injection molding). Unfortunately, creating iterations of prototypes for destructive and non-destructive testing to refine a new design is time consuming and economically unjustified. For this reason, many researchers are trying to apply numerical calculation methods to predict the response of a product to the forces that load it [9]. As a result, it is possible to significantly reduce experimental measurements, reduce the cost of manufacturing the product, and the time it can reach the customer [13]. In the case of medical applications, the latter factor may be one of the decisive factors, especially if the product is dedicated to a specific person.

Numerical models are used in medical engineering, for example, to predict the mobility of the tongue after surgery to remove neoplastic changes in tissues directly associated with this organ [14]. The finite element method is used to determine the stress arising in the human jaw [15], and the results of calculations can be used to design a jaw prosthesis [16]. In [17], the authors used the finite element method to predict the mechanical properties of scaffolds produced by the FDM/FFF method from PLA material subjected to compressive loads. The presented results showed a good correlation between the numerical experiment and measurements on real objects.

In addition, it should be noted that the literature lacks papers fully describing the methodology for obtaining the material data of products manufactured in the 3D printing technique and their application in numerical tests, especially medical devices. For example, [18] presented a method of conducting simulations and experimental research on open wrist orthoses (consisting only of the lower part of the shell). In this case, the force was applied to the extreme parts, from the palm side. This article does not deal with the material data used in the simulation. There are many articles describing simulation tests using the finite element method in which the results obtained were not compared with experimental tests. Some examples, where the mechanical parameters of pure material were adopted can be found in [19,20,21,22]; another example, where only the method of loading the orthosis simulation model is described, can be found in [23].

The literature review shows that it is reasonable to undertake research aimed at determining the suitability of numerical methods for determining the properties of products manufactured using the FDM/FFF method for the wrist orthosis. This product used in post-traumatic treatment should reach the patient in the shortest possible time. The numerical model should therefore meet two criteria. First, it should provide realistic results that can be used to design the geometry of the orthosis, enabling it to be quickly produced by the FDM/FFF method. Second, the calculations must be as short as possible so that, right after obtaining anthropometric data from the patient, it is possible to start the process of manufacturing an individualized orthosis.

A lot of descriptions of research works related to additive manufacturing are available in the literature. Their authors have prepared samples for determining the mechanical properties according to different standards by using different loading speeds, different sample production conditions, etc. Therefore, a direct comparison of the values of strength coefficients (particularly concerning bending strength) presented for the same material, but processed on different devices, may indicate significant differences in value, but should allow drawing consistent conclusions on the trend. For this reason, the authors of this work did not rely on literature data, but carried out their own experiments to provide adequate data for the analytical model.

## 2. Materials and Methods

### 2.1. Research Plan

The main goal of the presented work was to check whether it is possible to use simulation tools for the design of individualized orthoses manufactured additively using the FDM/FFF method. It was decided to check whether the properties of standard samples differed significantly from the properties of samples in the shape of a real object. For this purpose, the authors determined the mean values of density and modulus of elasticity for a series of tested samples. The orthoses should be characterized by the best possible strength properties while maintaining the lowest possible weight, enabling their comfortable use. Moreover, the production time of the orthosis should be minimized, so that the patient has access to the finished product within a few hours after scanning his limb. To achieve this goal, a series of experimental and simulation tests were planned.

The following activities were undertaken:

1. Production of samples: standard three-point bending samples, samples in the shape of the central part of the orthosis without openwork and with an openwork, samples in the shape of the entire orthosis;
2. Determination of the density of sample materials;
3. Bending tests;
4. Modeling and simulation of sample load (FEM); and
5. Comparison of the experimentally and simulated displacement values, determination of real value of elastic modulus on the basis of simulation results.

The purpose of the simulation model in this study was not to obtain as realistic a result as possible, but rather to acquire values necessary to calculate the real Young’s modulus for the geometries tested in the experiment. This will be used in further studies for carrying out more precise analyses, also taking into account properties such as the shear modulus, which was not considered here. In other studies, the authors considered the shear modulus in the strength calculations, but for objects of much simpler geometry [24]. Additionally, the material was assumed to be homogeneous; the anisotropy of properties, known in FDM products and investigated by the authors in previous studies [25,26] was not considered here, precisely because of the purpose of the simulation, as stated above.

The simulation model ensures the same geometry as the real samples, which allows the use of its results for the determination of real elastic modulus for any given sample. Knowing that the force used in the simulation (P_s_) is equal to the force obtained during the experiment (P_e_) and that the mass and geometric values are the same in the simulation and experiment, the real value of the elastic modulus (E_e_) can be determined according to Equation (1), derived directly from Hooke’s law.

E_e_ = (U_is_ × E_s_)/U_ie_, (1)

where:

E_e_—real value of elastic modulus (MPa);
E_s_—simulated value of elastic modulus (MPa);
U_ie_—experimentally obtained displacement value (mm); and
U_is_—simulated displacement value (mm).

The methodology and results of the work carried out are described in detail later in the article.

### 2.2. Manufacturing of Samples

In the tests, an unmodified FlashForge Creator Pro printer (FFF, Zhejiang Flashforge 3D Technology Co., Ltd., Jinhua, China) was utilized. The values of the most important operating parameters for the production process are described in Table 1. The 3D printer used a material especially dedicated by the manufacturers, however, the description of the mechanical properties of the material was not sufficient for use in the simulation tests. Material charts provided by material manufacturers usually contain data relating to the raw material processed by injection molding as was the case for the material used in these studies.

ABS (Spectrum Group Sp. z o.o., Pęcice, Poland) material was used for the production of all samples. It is characterized by high impact and temperature resistance as well as a glass transition temperature higher than other popular material, PLA, as well as a similar price [27]. All samples were made of one batch of material from the same manufacturer.

**Figure 1:**
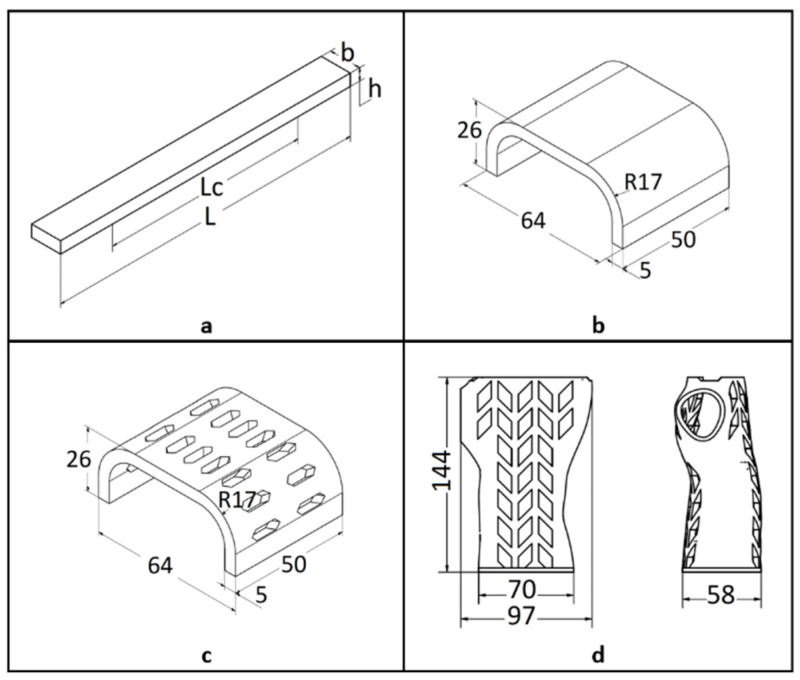
Dimensions of the test samples:(**a**) basic sample, iso view with the marked spacing of the supports of the testing machine: Lc = 100 mm, L = 64 mm, h = 4 mm, b = 10 mm.Samples in the shape of the central part of the orthosis: (**b**) full sample—orthosis fragment, (**c**) openwork sample—orthosis fragment, (**d**) orthosis-shaped sample (wall thickness of 4 mm).

In the first stage, test samples were created that were used for the three-point bending test (Figure 1a). The test samples were developed in accordance with the guidelines of EN ISO 178 [28]. Then, samples in the shape of the central part of the orthosis were produced in the full (Figure 1b) and openwork (Figure 1c) variants. The last (third) type of sample were orthosis-shaped samples without division into two shells (Figure 1d).

Linear filling (rectilinear) with a percentage of 15% was used for all the samples. The outer shell consisted of two contours 0.4 mm wide.

**Figure 2:**
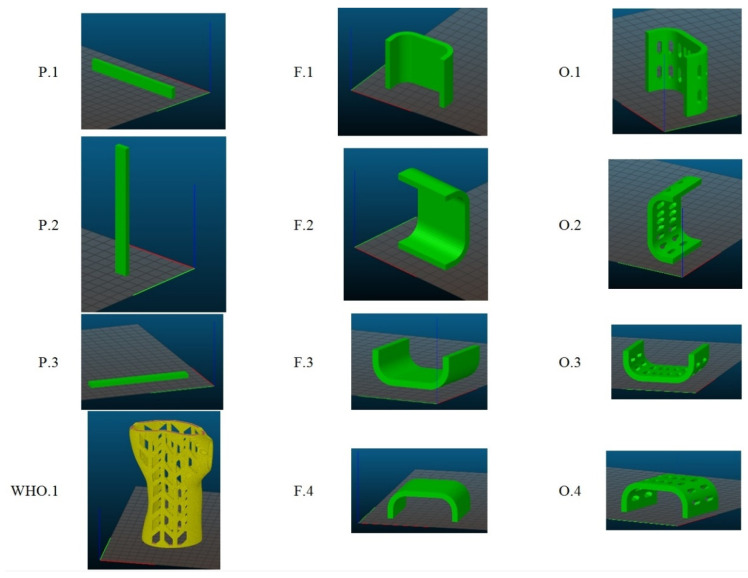
Designation of a series of samples and their arrangement in the working chamber.

The machine made a series of samples with different orientations in the printer’s working chamber (Figure 2). The series of three-point bending samples consisted of five samples; the series of samples in the shape of the central part of the orthosis and the whole orthosis consisted of three samples.

Table 2 shows the weights of individual samples and the average weight for a given series. The mass of the samples was determined using a precision balance by Radwag, model WTC 200 (top-loading balance, Radwag Wagi Elektroniczne, Radom, Poland). After production and mass measurement, the samples were kept under conditions limiting the influence of the environment on their mechanical properties.

Differences in mass between a particular series of samples of the same type resulted from the method of layering related to the orientation in the working chamber.

**Figure 3:**
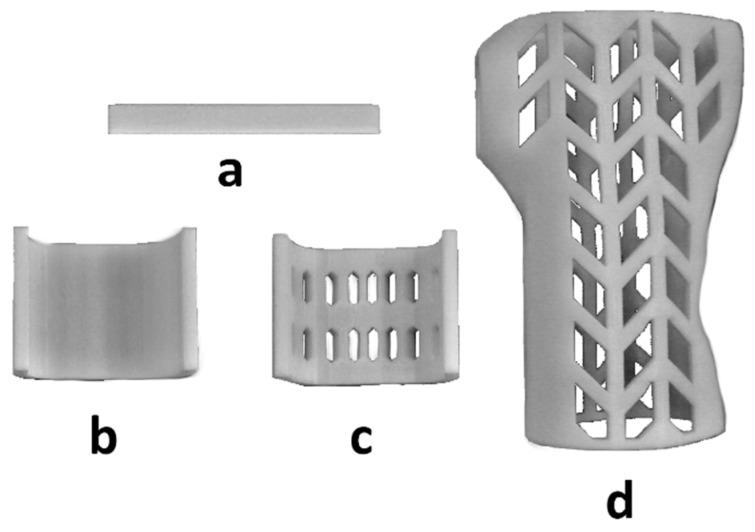
Manufactured samples, series: (**a**) P.1, (**b**) F.1, (**c**) O.1, (**d**) WHO.1.

Figure 3 shows examples of samples from the selected series. All manufactured samples were visually inspected in order to detect any structural anomalies resulting from errors that may have arisen in the manufacturing process. No errors were found that would disallow the use of a given sample in further parts of the study.

### 2.3. Methodology of Bending Tests

**Figure 4:**
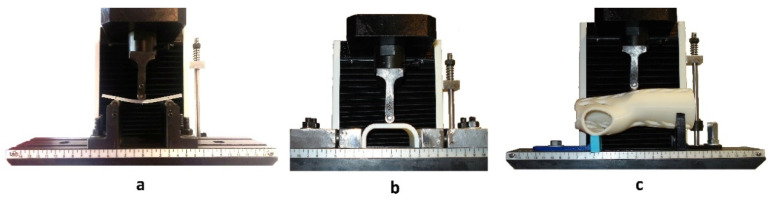
Three-point bending of the sample: view of the working tooling of the testing machine, bending of the sample: (**a**) basic, (**b**) in the shape of the central part of the orthosis, (**c**) in the shape of an orthosis.

Strength tests of the samples were carried out in accordance with ISO 178 on a Universal Testing Machine, model: WDW-5D-HS (Sunpoc, Guiyang, China), obtaining values of vertical displacement and force. The PN-EN ISO 178 standard [28] for three-point bending provides for the use of cuboidal samples with the ratio of height h to the spacing of supports L of L/h = 16, the tests assumed 64/4 = 16. Figure 4a shows the three-point method of sample loading [28].

Figure 4 shows a photograph of the samples during bending. The working mechanism of the grip of the testing machine for the orthosis and its fragments was prepared by the authors of the publication, and the main criterion of suitability was to ensure the stability of the position of the selected sample during the measurement.

The adopted method of loading the orthosis—three-point bending—is consistent with the actual way of loading the orthosis during its use.

### 2.4. Methodology of Finite Element Analysis

The ABAQUS 6.12-2 computational system (6.12-2, Dassault Systemes Simulia Corp., Rising Sun Mills, Providence, RI, USA), the standard module (statics) was used for the simulation tests. In order to obtain correct results in the modeling, higher-order spatial finite elements with a central node between the vertex nodes were used. This is due to the fact that in the case of a finite element mesh, the displacement value is calculated at each node, thus the more nodes – the more correct the obtained values. The results for two types of finite element mesh superimposed on the sample model were compared with the displacement (U_i_) results obtained during the experimental tests. Tetrahedral elements with 10 nodes on the element (C3D10) [29] were used to apply the mesh.

**Figure 5:**
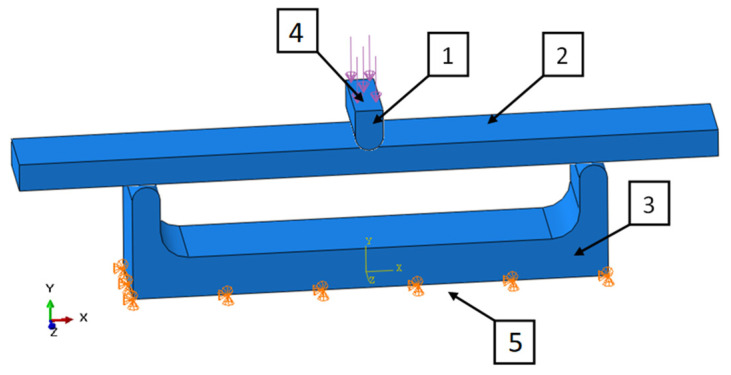
Simulation model of P series samples: (1) pressing element, (2) tested sample, (3) base, (4) place of application, (5) place of receiving force.

The simulation model for the basic samples was made in the form of an assembly, fully reproducing the working elements of the testing machine (Figure 5).

In order to check the possibility of shortening the computational time, the authors also loaded the base sample directly using the forces and boundary conditions. The obtained difference between the results for the sample indirectly loaded with the working elements and directly with the forces on the sample was less than 1%, and the reduction of the calculation time was threefold, from 3 to 1 min.

Modeling of the whole working system of the testing machine and the sample allows for a more faithful representation of the actual working conditions. The model takes into account the stiffness of the working tool by entering the steel material parameters. The applied contact algorithm calculates the actual contact area to which the force value is transmitted as pressure, which does not have a great impact on the quality, global results—deflection in the core (sample center), but only on local displacements of surface nodes.

The contact parameters between the surfaces of solids were determined using the command: “Mechanical—Normal Behavior: Constraint enforcement method: Default: Pressure-Overclosure: “Hard” Contact” in the ABAQUS software. Such a solution was used because the test was assumed static and the displacements minimal. Such an approach does not require taking friction into account. Influence of this model on the accuracy of the simulation results was considered as negligible by the authors.

**Figure 6:**
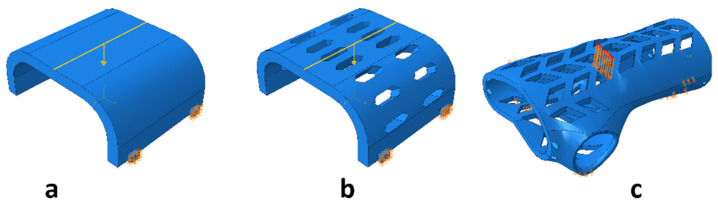
Boundary conditions and method of loading the sample in the shape of: the middle part of the orthosis: (**a**) full (series F), (**b**) with openwork (series O), (**c**) whole orthosis (WHO series).

In the case of the remaining samples, the working elements of the testing machine were not modeled, so it was limited to the application of appropriate forces and boundary conditions on the samples (Figure 6). The model with working elements requires the use of contact surfaces, which cause a significant extension of the calculation time compared to the static loading of the samples with forces in place operation of working tools and the assembly table (holder) of the testing machine. The time of calculations is important from the point of the possibility of their use in the production system for mass customization of products.

**Figure 7:**
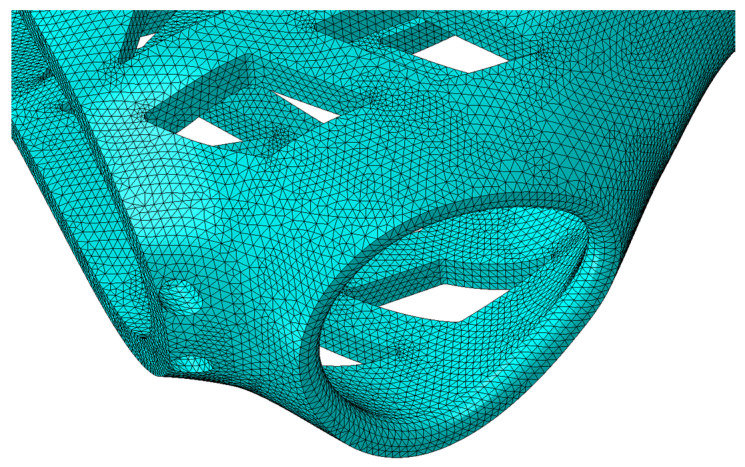
Finite element mesh superimposed on the test specimen in the shape of the entire orthosis of high geometric complexity (partial view).

View of a fragment of the finite element mesh of the orthosis model that accurately reflects the geometry of the orthosis curvatures is presented in Figure 7. The mesh parameters for all types of samples are presented in Table 3.

For the sake of clarity and to facilitate the calculations, all types of samples were given the same Young’s modulus of E_s_ = 1000 MPa and Poisson number = 0.38. The entered value of E, due to the adopted method of calculating the actual values of the modulus with the assumed homogenization of the material, is an indicative value used in accordance with Equation (1).

## 3. Results

### 3.1. Bending Test Results

**Figure 8:**
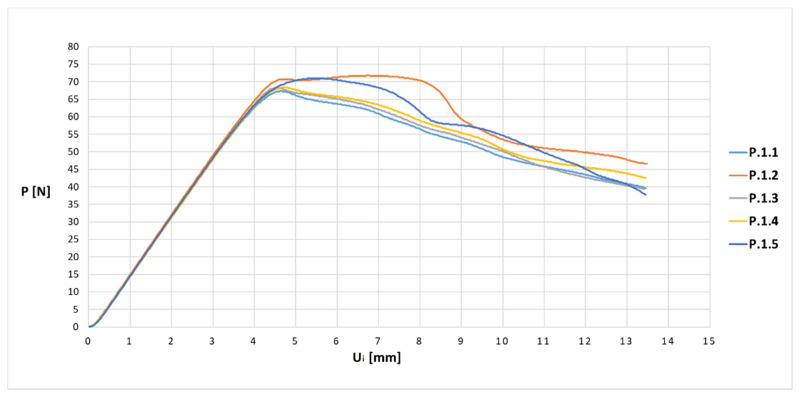
Force–displacement relationship diagram for samples from the P.1 series.

The experimental bending tests of the samples produced allowed us to obtain force (P)—displacement (U_i_) graphs for individual samples in the series. An exemplary diagram for a series of basic samples P.1 is shown in Figure 8.

Based on the experimentally obtained values of displacement, average values were determined. Table 4 summarizes the average values of the samples and the standard deviation of the population of the given series at the corresponding loading force.

As can be seen in Table 4, the obtained values of the standard deviation for a series of samples did not exceed the value of 0.05, which indicates a high repeatability of the manufacturing process.

**Figure 9:**
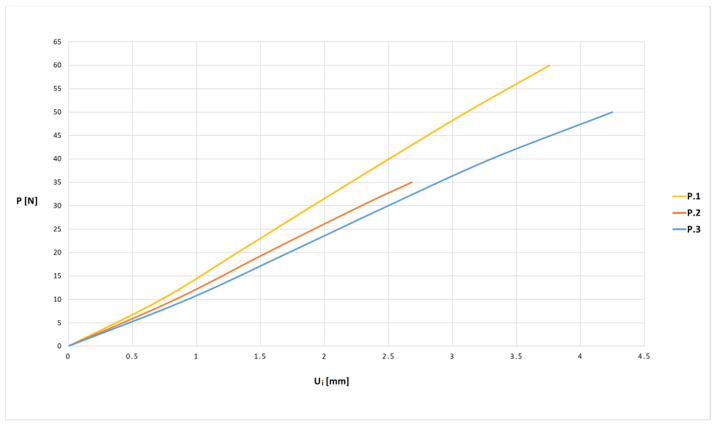
Force (P)–displacement (U_i_) dependence from average values for basic samples.

**Figure 10:**
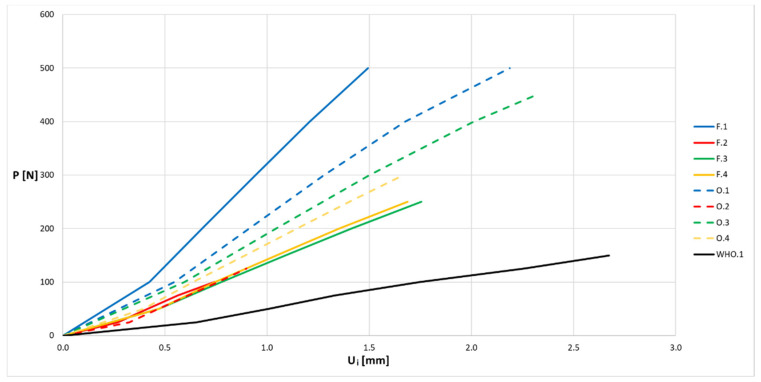
Force (P)–displacement (U_i_) dependence from average values for samples in the shape of the middle part of the orthosis and the entire orthosis.

The average values of displacement for all series of samples are presented in Figure 9 and Figure 10 in the form of force (P)–displacement (U_i_) diagrams.

When analyzing the obtained diagrams for average values (Figure 9 and Figure 10), it should be noted that full linearity was not obtained, which results from the internal structure: the overlapping of individual layers of the thermoplastic. The approximate linearity obtained should be combined with temperature fluctuations accompanying the position of the nozzle applying the material and variable thermal dissipation conditions affecting the bonding force at the boundary of layers and paths.

### 3.2. Finite Element Analysis Results

The comparison of the vertical displacement values obtained in the simulation tests using the FEM method (U_i_ = U_2_ = U_y_) is presented in Table 5.

The displacement U_i_ values presented in Table 6 are only of a reference nature and were used to determine the actual value of the elastic modulus.

### 3.3. Result Comparison

Table 6 summarizes and compares the obtained values of the density and modulus of elasticity of individual series of samples with the value for the series of the final product of the WHO orthoses.

The greatest convergence in the density value was obtained for the basic samples marked P.3, where the difference in relation to the orthosis was 1%. In the case of samples in the shape of the middle part of the orthosis with an openwork, the marking of series O.1 and O.2, the difference in density was small and was 2%. The remaining samples were characterized by values exceeding 7% and in the extreme case 15% in samples of the P.2 series.

As can be seen in the values of the modulus of elasticity (E) presented in Table 6, the orientation of the product in the working chamber (Figure 2) significantly affects its stiffness. For samples with lateral orientation, marking 1 (P.1., F.1. O.1.), the highest values of the elastic modulus were obtained. For basic samples (series P) and full orthosis fragments (series F), vertical orientation (orientation 2) allowed us to obtain higher stiffness than the flat orientation (orientation 3 and 4). However, for the fragments of orthoses with an openwork, the situation was the opposite.

The obtained values of the modulus of elasticity (E) between samples without openwork (P.1-3 and F.1-4) and with openwork (O.1-4 and WHO.1) differed significantly in terms of deformability in the linear range of their materials. In the case of basic samples (series marked with P), the difference in relation to the entire orthosis exceeded 100%, which suggests that the standardized three-point bending tests for materials manufactured with the FDM/FFF technique are not applicable. Due to the fact that the greatest convergence was obtained for samples in the shape of the central part of the orthosis with openwork, the O.3 series with 8% and the O.4 with 1% series, it seems reasonable that in the product design process, tests aimed at obtaining material properties should be carried out non-standardly, taking into account certain geometric assumptions such as the presence of openwork.

The highest values of the modulus of elasticity in individual types of samples were obtained for samples with the production orientation in the working chamber no. 1, that is, for samples from series P.1, F.1, and O.1. In these samples, the individual layers were applied perpendicularly to the force applied in the testing machine.

**Figure 11:**
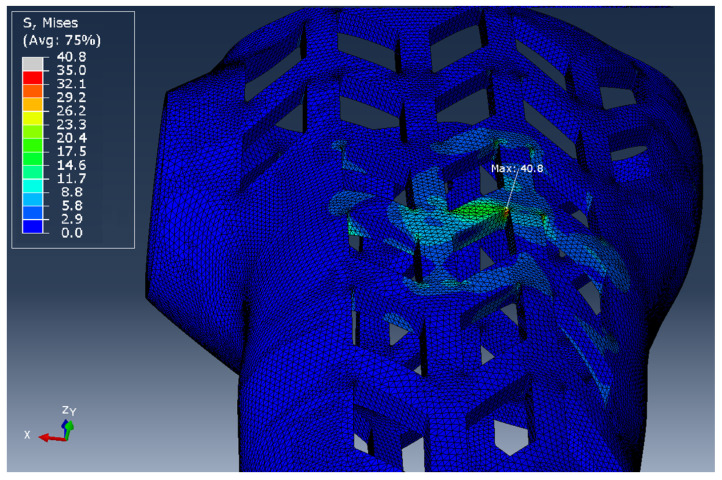
The obtained von Mises stress map on the WHO.1 orthosis model, visible local stress concentration of 40.8 MPa.

The presented simulation studies, with the full mapping of the model topology, take into account the stiffness resulting from the geometry of the tested object, but will not take into account changes in stiffness resulting from non-linearity of the material and disturbances related to the fracture process. Figure 11 shows the stress map obtained on the orthosis model loaded with the force P = 100 N. The stress values obtained on the FEM model, due to the assumed mechanical values, can only be used to detect the places of accumulation that initiate the fracture process. In further research, the authors will focus on the propagation of stress in the structure and their influence on the linearity disturbance.

**Figure 12:**
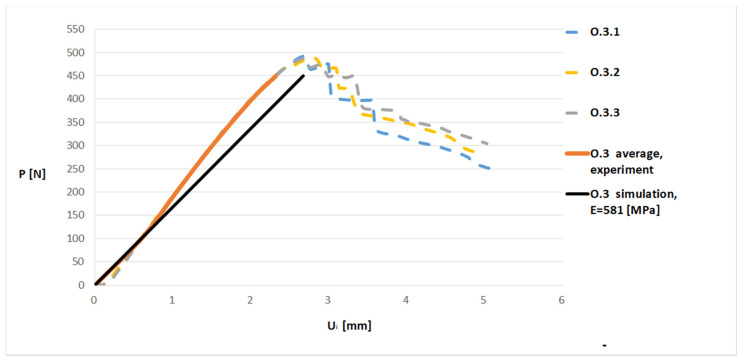
Force (P)–displacement (U_i_) dependence for the O.3 sample: juxtaposition of experimental and simulation results.

The introduction of the previously obtained value of the elasticity modulus to the simulation model caused the results obtained on the model to be similar to the experimental results (see Figure 12). The authors assumed in the simulations that the material was linear-elastic. Therefore, the lines for the average of the experiment and simulation shown in Figure 12 did not fully match. The authors intend to conduct research taking into account the non-linearity of the material in the future.

To check whether determined values of the elastic modulus produced more accurate simulation results, the value calculated for the samples from the O.3 series (E = 581 MPa) was entered into the model and it was loaded with the force P = 100 N. The obtained displacement value U_is_ was 0.595 mm, which was a 0.1% difference from the experimental results (U_ie_ = 0.596).

## 4. Discussion

The P.3 series of basic samples showed the greatest convergence with the WHO.1 complete sample in terms of density values. The WHO.1 density was obtained by entering the value of 1 [g/cm^3^] into the solid model and comparing the mass obtained from the model with the mass of the real object.

The density of products manufactured with the additive FDM/FFF method largely depends on their geometry. This was clearly visible for orthosis-shaped specimens with and without openwork. In openwork samples, the necessity to create many holes required the use of full layers of opening and closing in places under and above the holes. In the simplest terms, it can be said that the smaller the ratio of the product surface to its volume, the lower the actual density of the final product will be, under the same process conditions.

The standard deviation of density/mass is an indicator of the repeatability of the production. The smaller the deviation, the greater the repeatability. However, it should be remembered that the ABS input material in the form of a wire with a nominal diameter of 1.75 mm itself has some manufacturing tolerance. In the case of tests performed by the authors, it was assumed that the wire diameter deviation given by the manufacturer was equal to ±0.02 mm. This means that the wire cross-sectional area can vary within 4.5% of the nominal value. Other disturbances in the process not only affect the final weight of the product, but also its distribution in the product. In the presented research, the highest standard deviation was observed for the series O.1 and it was about 1.5% of the average weight of the products in the series. In the authors’ opinion, this is the value for which it can be concluded that the products in the series are repeatable. Additionally, thanks to visual inspection, the authors were able to rule out the appearance of discontinuities in the outer surface of the products, which could significantly change the indicators of the strength assessment of the product.

All the normative samples (P series) obtained the Young’s modulus values at least twice as high as for the orthosis. This means that the sample P is much less elastic, although in absolute terms, it is still only half of the Young’s modulus for a typical ABS material sample obtained by traditional injection molding. The material itself did not significantly change its Young’s modulus. The difference in recorded values should be attributed to a combination of process parameters, sample geometry, and its defects. Taking into account the sample density table and the table with the obtained Young’s modulus values, it can be stated that the first index absolutely cannot be used to determine the similarity of the strength properties. The sample P.3 has almost the same density as the orthoses, but twice the value of the Young’s modulus. In contrast, the O.3 series samples had an 8% difference in density compared to the orthosis, but had almost the same Young’s modulus. It is clearly visible that what matters is the distribution of the volume errors, rather than their total size, which determines the density. The significantly different sizes of the orthosis and standard samples also affect the temperature of the material during production at the interface of the next layer and adjacent paths. In the case of normative samples, the much shorter production time of a single layer meant that a stronger connection could have occurred at the layer boundary, which increased the stiffness of the entire sample.

The comparison of the results of the Young’s modulus for the samples of series F and O led to the conclusion that in the case of the use of openwork in products manufactured by the FDM/FFF method, it led to an increase, not a decrease, in the stiffness of the structure. Due to the nature of the process, especially, in the method of applying the contour material and filling the inside of the layer, in the case of an openwork orthosis, it worked similarly to an embossment on a sheet (e.g., a canister), increasing the stiffness of the entire structure. In the case of parts of the orthosis without openwork, the connection between the upper surface, to which the force was applied during the destructive tests, and the opposite surface, was made only of the material filling the interior of the middle layers. Due to the necessity to make the openwork, the extruded material appeared on the path of the force during the formation of the contour, which transfers the loads in the above-mentioned direction much better. For the F.2-4 series, the value of Young’s modulus was twice lower than that for the orthosis. Only for the F.1 series was the difference smaller, and resulted from the fact that the beam loading the orthosis from the top was positioned perpendicular to the production direction. This means that the outermost layers of the orthosis fragment, which have full filling, can bear the load, partially compensating for the significant weakness resulting from incomplete filling of the middle layers. It should be noted, however, that while an openwork orthosis requires a similar amount of material for its production as for an orthosis without an openwork, the production time is significantly longer. The shape of the openwork should also be selected so that it does not require the use of additional supports during its production.

Significant differences in the value of Young’s modulus between standard samples and samples in the shape of an orthosis or its central fragment mean that, unfortunately, in simulation studies, it is not possible to use the results of research works that are most often carried out with normative samples. Therefore, in the case of building a system for mass customization of a specific product produced additively by the FDM/FFF method, tests on a specific machine–material–product system are necessary. As shown by these tests, sufficient compliance can be achieved by using a properly prepared geometry of a product fragment.

The results presented in the work prove that in the case of material processed in the 3D printing technique on the same device, there is sufficient repeatability of the mechanical properties of the obtained products. This means that the process is stable and it is possible to use numerical simulation to estimate the strength properties for geometrically similar products.

It would be interesting in further studies to investigate the influence of the technological process parameters and the size of the orthosis on its mechanical properties by means of a sensitivity analysis. It is possible that the currently used technological parameters are not the best possible for a given orthosis manufacturing scenario (different sizes for children and adults) and that a better set could be found by implementing sensitivity analysis-based optimization, as did the authors in [30].

In the research, the authors wanted to use the existing FEM tool, which could be easily and quickly (out of the box) used with an automatic orthosis design system. It was important to check the degree of compliance of the FEM results with the real product and its simplified representations. An equally important evaluation criterion, in the case of the need to use FEM in mass customization, is the duration of calculations. The applied calculation model should therefore be as simple as possible. The model specific for the FDM/FFF process, also taking into account anisotropy (as well as geometric nonlinearity), is the subject of further research and the validity of its use with an automatic orthosis design system will be tested in the future.

## 5. Conclusions

The research results presented in the article confirm that the proposed method can be used for the strength assessment procedure of individualized orthoses during the design process, taking into account the influence of the FDM/FFF machine used. In the next step, the authors plan to create simulation models and their verification for complete orthoses of a selected group of patients.

The time required to perform numerical calculations using a dedicated unit of calculation is only a few percent of the time in which the product is manufactured. Moreover, better fitting of the product shape to the patient may reduce the manufacturing time associated with not having to extrude additional material. This means that for the final delivery time of the personalized orthosis to the patient, the use of numerical calculations may have a positive effect. Therefore, it is justified to carry out further works aimed at the implementation of the numerical simulation module in the systems of the mass customization of products.

The process of incremental manufacturing using the FFF method is described by many different parameters, which requires further studies supplementing the simulation library with new material data. From the point of production and use of orthosis-type products, in the opinion of the authors, it is particularly important to determine the influence of the temperature of the preceding layer on the value of Young’s modulus for the final product. It is also reasonable to check other thermoplastic materials and the simulation for the full geometry of the orthosis with validation on the real model.

## Figures and Tables

**Table 1:** Main parameters of 3D printing process.

Machine	Extrusion Temperature	Extrusion Speed	Layer Thickness	Material
FlashForge Creator Pro	240 °C	30 mm/s	0.25 mm	ABS

**Table 2:** Weight of the samples.

Series Designation	Sample No.	Weight of Samples [g]	Std. Deviation	Avg. Weight [g]
P.1	1	2.404	0.005	2.405
	2	2.405		
	3	2.396		
	4	2.407		
	5	2.413		
P.2	1	2.540	0.074	2.482
	2	2.396		
	3	2.549		
	4	2.386		
	5	2.539		
P.3.	1	2.187	0.009	2.191
	2	2.184		
	3	2.183		
	4	2.192		
	5	2.208		
F.1	1	11.288	0.078	11.210
	2	11.104		
	3	11.238		
F.2	1	11.468	0.061	11.403
	2	11.419		
	3	11.321		
F.3	1	11.107	0.021	11.120
	2	11.150		
	3	11.103		
F.4	1	11.416	0.048	11.484
	2	11.523		
	3	11.514		
O.1	1	10.741	0.155	10.641
	2	10.423		
	3	10.760		
O.2	1	10.591	0.010	10.606
	2	10.612		
	3	10.614		
O.3	1	11.218	0.016	11.235
	2	11.231		
	3	11.257		
O.4	1	11.251	0.062	11.180
	2	11.189		
	3	11.099		
WHO.1	1	49.272	0.045	49.332
	2	49.342		
	3	49.382		

**Table 3:** Parameters of the applied finite element mesh on the test samples.

Sample, Series	Shape of Element	Type	Global Size	Amount of Nodes	Amount of Elements
Basic, P	Tetrahedral	C3D10	1 mm	42,567	27,478
Fragment, F	Tetrahedral	C3D10	1.25 mm	110,629	72,696
Fragment, O	Tetrahedral	C3D10	1.25 mm	104,163	66,515
Entire orthosis, WHO	Tetrahedral	C3D10	1.00 mm	1,219,383	807,520

**Table 4:** The obtained average values of the U_i_ displacement from the P series of samples.

Series Designation	Obtained Average Value U_i_ [mm]	Std. Deviation	Loading Force [N]	Qty of Samples
P.1	0.728	0.012	10	5
P.2	0.837	0.030		
P.3	0.933	0.045		
F.1	0.424	0.029	100	3
F.2	0.742	0.044		
F.3	0.777	0.019		
F.4	0.744	0.008		
O.1	0.547	0.029		
O.2	0.759	0.028		
O.3	0.596	0.008		
O.4	0.638	0.045		
WHO.1	1.747	0.083		

**Table 5:** U_2_ vertical displacement for all sample series.

Series Designation	P Force Value [N]	Obtained U_i_ Displacement [mm]	Result as a Colorful Map	Approx. Computing Time [s]
P	10	1.028	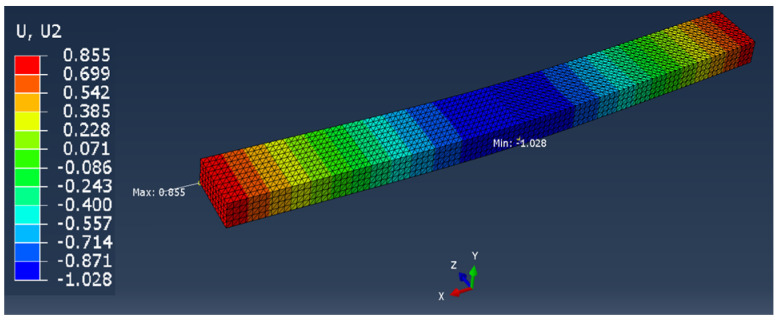	60
F	100	0.192	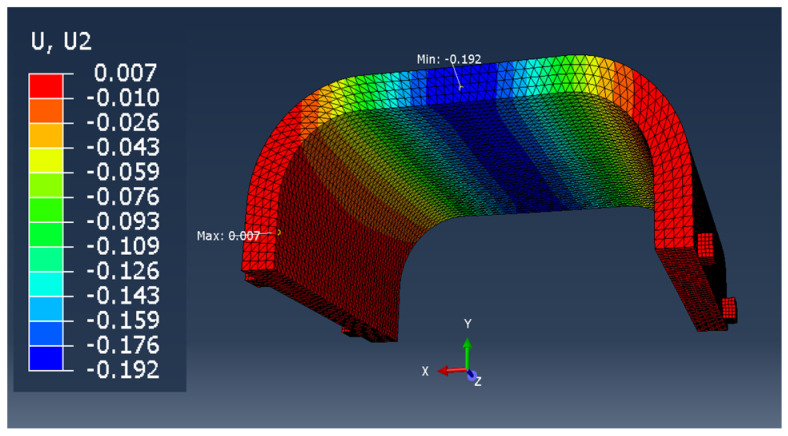	150
O	100	0.346	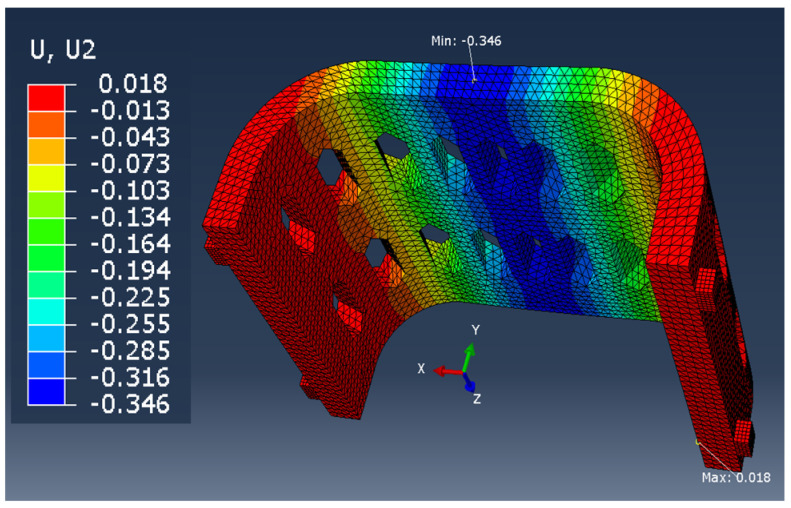	150
WHO	100	0.937	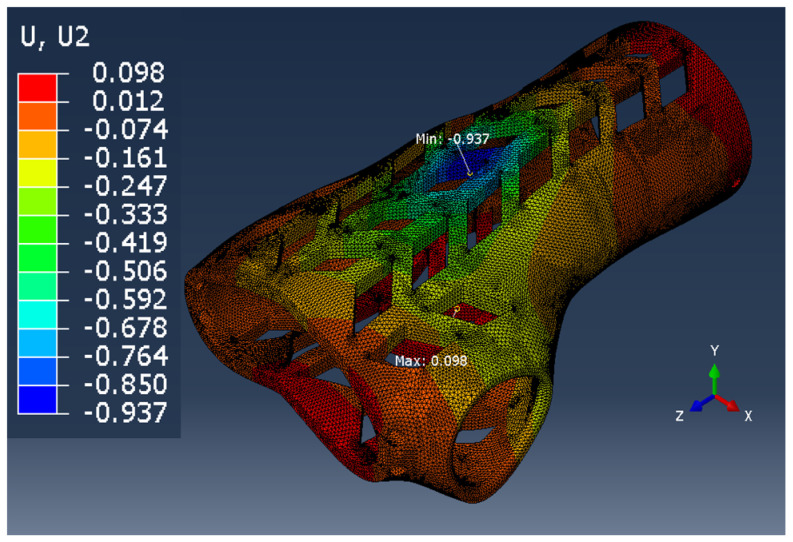	600

**Table 6:** Density and modulus of elasticity obtained for the samples.

Designation	Average Density ρs [g/cm^3^]	Deviation from Series WHO.1 [%]	Obtained E Modulus [MPa]	Deviation from Series WHO.1 [%]
P.1	0.601	11	1423	165
P.2	0.621	15	1238	131
P.3	0.548	1	1110	107
F.1	0.479	12	453	16
F.2	0.488	10	259	52
F.3	0.475	12	247	54
F.4	0.491	9	258	52
O.1	0.554	2	633	18
O.2	0.552	2	456	15
O.3	0.585	8	581	8
O.4	0.582	7	543	1
WHO.1	0.542	0	536	0
